# Neuroticism as a Moderator of Direct and Mediated Relationships Between Introversion-Extraversion and Well-Being

**DOI:** 10.5964/ejop.v12i1.985

**Published:** 2016-02-29

**Authors:** Daniela Fadda, L. Francesca Scalas

**Affiliations:** a Department of Education, Psychology, Philosophy, University of Cagliari, Cagliari, Italy; Department of Psychology, University of Western Ontario, London, Ontario, Canada

**Keywords:** subjective well-being, self-esteem, extraversion, neuroticism, adolescence

## Abstract

Among personality traits, extraversion has received major theoretical and empirical attention as predictor of subjective well-being (SWB), whereas the role of emotional stability-neuroticism has been partially neglected. The present study aims to study the role of neuroticism in the relationship between introversion-extraversion and SWB. In particular, we explored if the trait of neuroticism moderates the relationships between introversion-extraversion and SWB dimensions (Satisfaction with life, Mastery, Vigour, Social Cheerfulness), directly and by mediation of self-esteem. Indeed, previous studies have suggested that self-esteem is positively associated with high extraversion and low neuroticism and that it positively mediates the relationship between SWB and personality traits in adolescents. For this purpose, a sample of high school students (N = 1173) completed the Oxford Happiness Inventory, the Rosenberg Self-Esteem Scale and the Big Five Questionnaire. In a latent variable model, we examined the interaction effects (direct and indirect) of extraversion and neuroticism on SWB dimensions. Our results showed that the nature of differences between introverts and extraverts on SWB could be related to the level of neuroticism in relation to Satisfaction with life. Moreover, self-esteem mediated the relationship between personality traits and SWB. In particular, mediated moderation effect analysis showed that self-esteem mediates completely the relationship between the interaction term (extraversion x neuroticism) and Mastery, and partially the relationship with Satisfaction with life. Moreover, moderated mediation effect analysis showed that high levels of neuroticism moderate the effect of extraversion on Satisfaction with life and Mastery through the mediation of self-esteem. In conclusion, our results suggest that although extraversion has a cardinal role on SWB dimensions related to Vigour and Social Cheerfulness, neuroticism and the mediating role of self-esteem should more properly considered in relation to Satisfaction with life and Mastery.

Subjective well-being (SWB) is a broad term that encompasses the various ways people evaluate their lives. From a holistic perspective, SWB is characterized by affective and cognitive factors including concepts such as life satisfaction, satisfaction with domains such as work and health, pleasant emotions, low levels of unpleasant emotions, feelings of fulfilment and meaning (e.g., Pavot & Diener, 2008). According with Seligman (2002, 2011) SWB is a multifaceted construct that cannot be reduced to overly simplistic ideas.

A multidimensional perspective seems to be appropriate in the study of SWB across the lifespan, and particularly in adolescence (e.g., Kirschman, Johnson, Bender, & Roberts, 2009; Lerner, 2009). Among the various contributions to the holistic perspective on SWB in adolescence, this study takes into account the multidimensional model of Argyle et al. (Argyle, 2001; Argyle, Martin, & Crossland, 1989). The authors have extended their initial perspective on happiness (composed of cognitive and affective components), developing a multidimensional conception of SWB that includes several elements related to self-fulfilment, optimism, sense of control, positive relations with others, purpose in life, personal growth and physical health. Transitioning from Argyle's perspective, we can consider five dimensions of SWB. Satisfaction with life is an important factor in the prevention of psychopathology (Huebner, Suldo, Smith, & McKnight, 2004) and it mediates the impact of stressful life events and parenting behavior on adolescent risk behavior (McKnight, Huebner, & Suldo, 2002). Mastery included capacities of self-fulfillment that are increasingly relevant during adolescence because they are associated with the development and acquisition of behavioral and emotional autonomy (Steinberg & Silverberg, 1986). Vigour can be defined as the energy required to engage in activities and achievements (Mahon & Yarcheski, 2002). Social interest and Social cheerfulness relate to interest in other individuals and fun in social groups are relevant in the promotion of social development (Bonaiuto, Perucchini, & Pierro, 1997; Meeus, 1994).

## Extraversion and SWB

Extraversion is often considered the personality trait most relevant to the study of SWB (e.g. Diener, Suh, Lucas, & Smith, 1999). Eysenck (1983) defined happiness as stable extraversion, and argued that it appears to be related to easy sociability and a predisposition to enjoyable interactions with other people. Extraversion predisposed young people to have favorable life events, especially in the domains of friendship. These in turn led to a high level of positive SWB and to increments in extraversion (Headey, Glowacki, Holmstrom, & Wearing, 1985; Pavot, Diener, & Fujita, 1990; Watson, Clark, McIntyre, & Hamaker, 1992). Costa and McCrae (1980) supported the long-term effect of extraversion on SWB in an examination of the relationship between personality measures and levels of SWB obtained ten years later. Furthermore, Costa, McCrae, and Norris (1981) found this relationship so robust that extraversion could predict SWB over a 17-year period.

Despite the relationship between extraversion and SWB, Hills and Argyle (2001a) found that a substantial minority of subjects can be classified as “happy introverts”. These individuals prefer solitude, intimate relations and involvement in introspective activities. To explain this finding, the authors suggested that the extraversion trait affects SWB by a specific mechanism that reflects the ways individuals choose to attain happiness. Introverts may not derive much satisfaction from gregarious situations, but they could be no less open to other kinds of happiness. Introverts may establish individual affiliative relationships with a few special friends, they may have highly satisfying leisure activities and they may also enjoy an intense inner life, based on intellectual, musical or religious activities, which give them much to think about without the need to rely on other people (Storr, 1988).

Eysenck (1967) states that the degree of introversion-extraversion is determined by the level of activity in the ascending reticular activating system (ARAS). The extravert person is postulated to have a low level of arousal, so he/she seeks out more intense stimuli; introverts are characterized by high levels of activity and so will find their optimal level of arousal at a much lower level of stimulation.

Moreover, in Hills and Argyle’s (2001a) study, the variables more strongly associated with happiness, life regard and self-esteem, do not have a dominant social connotation. Both introverts and extraverts can achieve high level of happiness, and comparisons between the portioned groups of happy extraverts and introverts reveal no significant differences in self-esteem. The authors concluded: “The evidence that low introversion-extraversion scores are not inimical of happiness is unequivocal. This suggests that introversion-extraversion may be more of an instrumental variable which reflects rather than determines how individuals choose to attain life satisfaction and happiness” (Hills & Argyle, 2001a, p. 607).

## Neuroticism as Moderator of the Relationship Between Extraversion and SWB

According to Eysenck’s theory of the biological bases of personality traits (1967), the introversion-extraversion dimension can be dichotomized in four groups based on neuroticism predisposition: Stable extraverts (low arousal), unstable/neurotic extraverts, stable introverts (both moderate arousal), and unstable/neurotic introverts (high arousal). Stable extraverts are described as playful, easygoing, carefree, sociable and contented. Unstable extraverts are considered quickly aroused, egocentric, hot headed and histrionic. Stable introverts are calm, reasonable, controlled ad persistent. Unstable introverts are regarded as anxious, serious, worried, suspicious and thoughtful (Eysenck, 1967). Considered their arousal level, unstable introverts would be expected to avoid excessive external stimulation. Whereas stable extraverts would be expected to actively pursuit external stimulation. In a sample of preadolescents, Young and Bradley (1998) found that the unstable introverts regarded themselves as less happy and popular than other subjects. The authors suggested that it is not simply introversion that determines negative social consequences, neuroticism also needs to be considered. Therefore, unstable introverts may be more likely to suffer from maladjustment than stable introverts.

In general, individuals higher in neuroticism are more unhappy than those lower in neuroticism (e.g., Costa & McCrae, 1980; DeNeve & Cooper, 1998; Diener, Oishi, & Lucas, 2003; Diener, Suh, Lucas, & Smith, 1999; Steel, Schmidt, & Shultz, 2008). In a meta-analysis of the relationships between 137 distinct personality constructs and SWB, DeNeve and Cooper (1998) found that emotional stability was the strongest predictor of SWB. Moreover, Hills and Argyle (2001a, 2001b) suggested that emotional stability might be more important to individual SWB than extraversion and its component of sociability.

Studies in which both extraversion and neuroticism are included as independent variables reveal that the effect of neuroticism on SWB normally outweighs the effect of extraversion (Brebner, Donaldson, Kirby, & Ward, 1995; David, Green, Martin, & Suls, 1997; DeNeve & Cooper, 1998; Pavot, Fujita, & Diener, 1997; Ryan & Frederick, 1997). Vittersø (2001) found in a sample of adolescents that, when controlled for emotional stability, the effect of extraversion is attenuated or disappears altogether (see also: Hotard, McFatter, McWhirter, & Stegall, 1989; Pavot et al., 1997). Moreover, Vittersø and Nilsen (2002) found that emotional stability is a predictor of SWB more important than introversion-extraversion; indeed, emotional stability explains eight times as much of the SWB variance as does extraversion.

## Self-Esteem as Mediator of the Relationship Between SWB and Personality Traits

High extraversion and low neuroticism have been positively associated with self-esteem in adult and adolescent samples (Erdle, Gosling, & Potter, 2009; Robins, Tracy, Trzesniewski, Potter, & Gosling, 2001), as well as in cross-cultural studies (Fickova, 1999). For example, Erol and Orth (2011) found that during adolescence and early adulthood “at each age, emotionally stable, extraverted, and conscientious individuals experienced higher self-esteem than emotionally unstable, introverted, and less conscientious individuals did” (Erol & Orth, 2011, p. 7).

Self-esteem is one of the strongest predictors of SWB and psycho-social adaptation (Diener, 1984; Neto, 2001; Rosenberg, 1965). Poor self-esteem has been associated with indices of poor adjustment (Bosacki, Dane, Marini, & Youth Lifestyle Choices - Community University Research Alliance, 2007), whereas high self-esteem has been associated with SWB (DeNeve & Cooper, 1998; Diener, 1984; Rosenberg, Schooler, Schoenbach, & Rosenberg, 1995), despite differences in culture and/or nationality (Diener & Diener, 1995). Studies that have examined the relationship between self-esteem and SWB in adolescence have identified patterns similar to adults, with a strong association between the two variables (Cheng & Furnham, 2004).

Previous studies have suggested that self-esteem positively mediates the relationship between SWB and personality traits in adolescents. For example, Furnham and Cheng (2000) using path models, found that extraversion exhibited direct and indirect predictive power, whereas neuroticism predicted SWB only through the mediation of self-esteem. Subsequently, Cheng and Furnham (2003) found that only self-esteem had direct predictive power on the SWB total score. Moreover, because extraverts (compared with introverts) tended to have higher self-esteem, and individuals with high levels of neuroticism (compared with stable individuals) tended to have lower self-esteem, the authors suggested that extraversion and neuroticism predicted SWB through self-esteem. Support to the mediating role of self-esteem derives from a recent study (Fadda, Scalas, & Meleddu, 2015) based on measured variables (i.e., scale scores) confirming that self-esteem is not only a direct predictor of SWB dimensions, but also a crucial mediator of the relationship between personality traits and SWB. Fadda et al. (2015) found that self-esteem positively influenced several SWB dimensions (in particular the sense of Mastery and self-fulfillment, but also Satisfaction with life, Vigour and Social cheerfulness) and that both extraversion and neuroticism affect all SWB dimensions directly and with exception of Social interest, through the mediation of self-esteem.

### Interactional Model of Personality

It should be noted that, although the relationships between self-esteem, extraversion, neuroticism and SWB are powerful, at the moment it is still not possible to clearly provide the direction of causality (Diener et al., 1999; McNiel & Fleeson, 2006).

Within this context, it appears useful to consider the interactional models of personality (Endler & Magnusson, 1976; Mischel & Shoda, 1999), which focuses on the circular process that takes place in person-situation interaction. According to these models, the individual perception of SWB will be determined by a complex pattern of relations between cognitive and affective units that will be activated by particular configurations of features present in situations. The stable personality structure of the individual determines the relationships between the types of situations encountered and the cognitive, affective and behavioral responses (Shoda & Mischel, 2000).

Although interactional models of personality are recursive and would require a longitudinal study that can follow their development over time, in the present investigation we were confined to a static correlational model. The examination of this model is possible through the use of SEM able to define the structure of the complex network of direct and indirect links between the variables.

## Present Study

The present study aims to study the role of neuroticism in the relationship between introversion-extraversion and dimensions of SWB (Satisfaction with life, Mastery and self-fulfillment, Vigour and Social cheerfulness). Our literature review (Hills & Argyle, 2001a, 2001b; Vittersø, 2001; Young & Bradley, 1998) showed that neuroticism is implied in individual SWB determination, and suggested that its role might be more critical than introversion-extraversion, particularly in young people. Therefore, the role of neuroticism needs to be further explored.

In particular, we explored if the trait of neuroticism moderates the relationships between introversion-extraversion and dimensions of SWB, directly and by mediation of self-esteem. Previous studies (Cheng & Furnham, 2003; Fadda et al., 2015; Furnham & Cheng, 2000), supported the mediating role of self-esteem in adolescents; however, these study were confined to observed variables, which do not take into account measurement error.

The present study addresses limitations from previous studies considering the interconnections between personality traits (extraversion, neuroticism and their interaction), self-esteem, and SWB within a single latent model.

We hypothesize (Hypothesis a) that the trait of neuroticism moderates the relationships between introversion-extraversion and SWB. In particular, moving from Eysenck’s (1967) theory of cortical arousal, we expect that the relations of introversion-extraversion with SWB will be different in individuals characterized by different levels of neuroticism. Specifically, we expect a1) that between extraverts, unstable individuals will show lower levels of SWB than stable extraverts and more similar to stable introverts (Eysenck, 1967). Moreover, we expect a2) that unstable introverts will show the lowest levels of SWB (Hills & Argyle, 2001a, 2001b; Young & Bradley, 1998). Finally, we expect (Hypothesis b) that the neuroticism of unstable introverts and extraverts might negatively impact self-esteem, which in turn will decrease SWB (Erol & Orth, 2011).

## Methods

### Participants

The present study investigates SWB in adolescence. Since in Italy attending high school is compulsory for adolescents, at least up to the age of 16, high school was an ideal context for questionnaires administration. Thus, for the recruitment of participants to the study and in order to have a representative sample of high-school Italian students, we contacted 20 high-schools from different fields of study (e.g., scientific, humanistic, technical). The convenience sample, balanced for gender, consists of 1173 adolescents (580 males and 593 females) aged between 14 and 20 years (*M* = 16.78; *SD* = 1.70). Consistent with the general Italian school population, the sample was homogeneous in terms of race (Caucasian) and cultural background (all students spoke Italian as their native language).

### Measures

To measure SWB we used the revised Oxford Happiness Inventory (OHI, Argyle et al., 1989; Italian version: Meleddu, Guicciardi, Scalas, & Fadda, 2012). The OHI consists of 29 items on a 4-point response scale. Each item invites the respondent to choose one of four sentences formulated to reflect incremental statements. For example: 1 = *I do not feel happy*; 2 = *I feel fairly happy*; 3 = *I am very happy*; 4 = *I am incredibly happy*. Using exploratory structural equation modelling (ESEM; Asparouhov & Muthén, 2009; Marsh et al., 2009), Meleddu et al. (2012) validated the Italian version of OHI in a sample of adolescents. A five-factor structure (Satisfaction with life, Mastery, Vigour, Social Interest and Social Cheerfulness) with strong measurement invariance over gender was found and adequate internal consistency for most of the scales (total scale, α = .90; Satisfaction with life, α = .81; Mastery, α = .80; Social Cheerfulness, α = .74; Vigour, α = .67; Social Interest, α = .65) could be demonstrated. In the present sample, Cronbach’s alpha was good for Satisfaction with life (6 items; α = .77), Mastery (10 items; α = .75) and Social Cheerfulness (5 items; α = .74), sufficient for Vigour (5 items; α = .63) and poor for Social Interest (2 items; α = .57). To assess internal consistency, we used also the omega coefficient, which has been shown to be a more sensible index in comparison to Cronbach’s alpha (e.g., Graham, 2006; Zinbarg, Revelle, & Yovel, 2007; Zinbarg, Revelle, Yovel, & Li, 2005). Omega coefficient was good for: Satisfaction with life (ω = .98), Mastery (ω = .92), Vigour (ω = .93), Social Cheerfulness (ω = .97), and sufficient for Social Interest (ω = .75). Given the psychometric limits of the scale, and a lack of an effect of self-esteem on Social Interest in previous studies (Fadda et al., 2015; Meleddu et al., 2012), we will not take into account this variable in the present investigation.

To measure self-esteem we used the Rosenberg Self-Esteem Scale (RSE, Rosenberg, 1965; Italian version: Prezza, Trombaccia, & Armento, 1997). RSE is composed of 10 items with a 4-point Likert (from 1 = *strongly disagree* to 4 = *completely agree*) response scale. The Italian version showed adequate internal consistency and test-retest reliability values, as well as good construct validity and a factorial structure, similar to that found in other countries (Prezza et al., 1997). In the present sample, internal consistency was α = .83.

To measure personality traits we administered the Extraversion and Neuroticism scales of a Italian questionnaire for the measurement of the Big Five Factor Model (Big Five Questionnaire, BFQ; Caprara, Barbaranelli, & Borgogni, 1993), with a 5-point response format (from 1 = *absolutely false* to 5 = *absolutely true*). The BFQ (that included also Agreeableness, Conscientiousness, and Openness to Experience scales) showed good factorial structure and adequate internal consistency. In the present sample, we found adequate internal consistency for both scales (α = .74 for Extraversion and α = .84 for Neuroticism).

### Procedure

For every school, we preliminarily obtained informed consent from parents using an opt-out procedure, so that only parents not interested in the study would have to return back the signed form. Participants anonymously completed the questionnaires within a larger battery during their regular school time. It took about 20 minutes to complete the questionnaires considered in the present study. The guarantee of confidentiality was stressed and only 12 students decided not to participate in the study. Because self-report measures may be subject to distortions, and in particular, demand characteristics can influence reports (Nichols & Maner, 2008; Orne, 1962), the questionnaires were administered in similar conditions (in class context during school hours) by the same researcher, instructed to provide the same information (e.g., self-presentation, instructions, overall goals of the study, anonymity and other ethical issues) to all participants.

### Analyses

In order to proceed with the statistical analyses all the variables were standardized. Hypotheses testing was carried out with structural equation models (SEM*;* Bollen, 1989) using the Mplus program (5.2. version). Self-esteem was defined by a trait substantial factor, along with two method factors (Marsh, Scalas, & Nagengast, 2010; Quilty, Oakman, & Risko, 2006). In line with the Italian validation (Caprara et al., 1993), the traits of extraversion and neuroticism were assessed through two latent constructs with two indicators each (respectively: Dynamism and Dominance; Lack of emotional control and Lack of impulse control). For SWB, in line with the Italian validation of the OHI (Meleddu et al., 2012), we used an ESEM approach with a four-factor structure (Satisfaction with life, Mastery, Vigour, and Social Cheerfulness).

To evaluate results, several fit indices were taken into account: Chi-square, Comparative Fit Index (CFI), Tucker–Lewis Index (TLI), Root-Mean-Square Error of Approximation (RMSEA). CFI and TLI vary from 0 to 1, with values close to 1 indicating a better fit. For RMSEA, values lower than .06 indicate a potentially better fit (Hu & Bentler, 1999), although no golden rules have been established (Chen, Curran, Bollen, Kirby, & Paxton, 2008; Marsh, Hau, & Wen, 2004).

**Figure 1:**
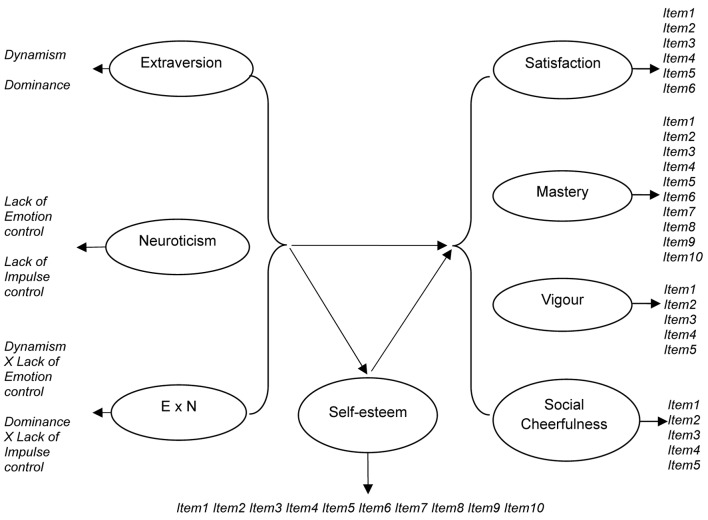
Conceptual path diagram. *Note.* The arrows indicate the causal direction of influences.

Our latent model (see Figure 1) is characterized by correlations between the exogenous variables, introversion-extraversion and emotional stability-neuroticism; direct effects of personality traits and self-esteem on SWB dimensions, and indirect effects of traits on SWB mediated by self-esteem.

To test the moderating effects of emotional stability-neuroticism, we have included in the model a latent interaction term between extraversion and neuroticism, using the product of indicators method (Marsh, Wen, & Hau, 2004). Moreover, to better understand the mediating role of self-esteem, based on our results, we performed post hoc tests of mediated moderation (e.g., Baron & Kenny, 1986) and moderated mediation (Cheung & Lau, 2015).

It is not uncommon for hypotheses about moderation and mediation relationships to occur in the same context. In the models in which interaction effects are mediated, the effect is termed mediated moderation (Baron & Kenny, 1986). Baron and Kenny (1986) provided indications to compute how much of the total effect of the moderator is due to the mediator. Complete mediation is the case in which the predictor (the interaction term in our models) no longer affects the outcome variable (e.g. Mastery) after the mediator (self-esteem) has been controlled. Partial mediation is the case in which the path from the predictor to the outcome is reduced in absolute size but is still different from zero when the mediator is introduced.

When an indirect effect is moderated by at least one moderator variable, the effect is termed moderated mediation (James & Brett, 1984). In moderated mediation models, researchers investigate whether the strength of an indirect effect depends on the value of the moderator, resulting in a conditional indirect effect (Preacher, Rucker, & Hayes, 2007). The index of moderated mediation is the change of the indirect effect of the predictor (e.g. extraversion) on the outcome variable (e.g. Satisfaction with life and Mastery) through the mediator (self-esteem) for a unit change in the moderator (neuroticism). It should be noted that the indirect effects at different levels of the moderator are statistically and significantly different when the confidence interval of the moderated mediation index does not include zero (Cheung & Lau, 2015). Cheung and Lau (2015) recommend to use bootstrapping in testing moderated mediation effects in latent interaction models. With bootstrapping, the sampling distribution of the moderated mediation effect is first estimated non-parametrically, then the information from the bootstrap sampling distribution is used to construct a confidence interval for the moderated mediation effect (Preacher et al., 2007). Because this analysis is still not available for ESEM, for these tests we used as outcome variables the corresponding factors specified with CFA.

## Results

### Measurement Model

As a preliminary test, we examined the measurement model based on correlated latent variables with no structural regression paths nor the interaction variable. Considering the complexity of the model, it showed an adequate fit to the data (χ^2^ = 1532.87; *df* = 642; *p* < .01; CFI = .92; TLI = .91; RMSEA = .034). In Table 1, we report the correlations among the variables^i^.

**Table 1:** Correlations Between the Variables in the Measurement Model.

Variable	1	2	3	4	5	6
1. Extraversion	—					
2. Neuroticism	-.29**	—				
3. Self-esteem	.61**	-.52**	—			
4. Satisfaction with life	.40**	-.39**	.57**	—		
5. Mastery	.51**	-.56**	.79**	.50**	—	
6. Vigour	.53**	-.18	.32**	.37**	.40**	—
7. Social Cheerfulness	.60**	-.25**	.42**	.46**	.45**	.31**

**p < .01.

### Structural Model

To test our hypotheses, we examined a model including all the latent constructs, as well as the latent-variable interaction between extraversion and neuroticism, and the structural paths among the variables (see Figure 1). Despite its complexity, the model showed adequate fit indices (χ^2^ = 1610.95; *df* = 710; *p* < .01; CFI = .92; TLI = .90; RMSEA = .033).

#### Direct Effects on SWB

Regarding direct influences of traits on SWB dimensions, main effects were in line with previous studies (see Table 2). Extraversion promoted Vigour and Social Cheerfulness; neuroticism negatively influenced Mastery. Moreover, self-esteem positively influenced Satisfaction with life and Mastery.

**Table 2:** Direct and Indirect Effects on SWB Dimensions.

Variable	Satisfaction with life	Mastery	Vigour	Social Cheerfulness
Direct effects				
Extraversion	.08	.12	.62**	.56**
Neuroticism	-.04	-.21**	-.05	-.09
Interaction	-.33**	-.01	.09	.09
Self-esteem	.56**	.62**	-.13	.00
Indirect effects mediated by self-esteem				
Extraversion	.27**	.29**	-.06	.00
Neuroticism	-.24**	-.27**	.06	.00
Interaction	.11*	.13*>	-.03	.00

*p < .05. **p < .01.

**Figure 2:**
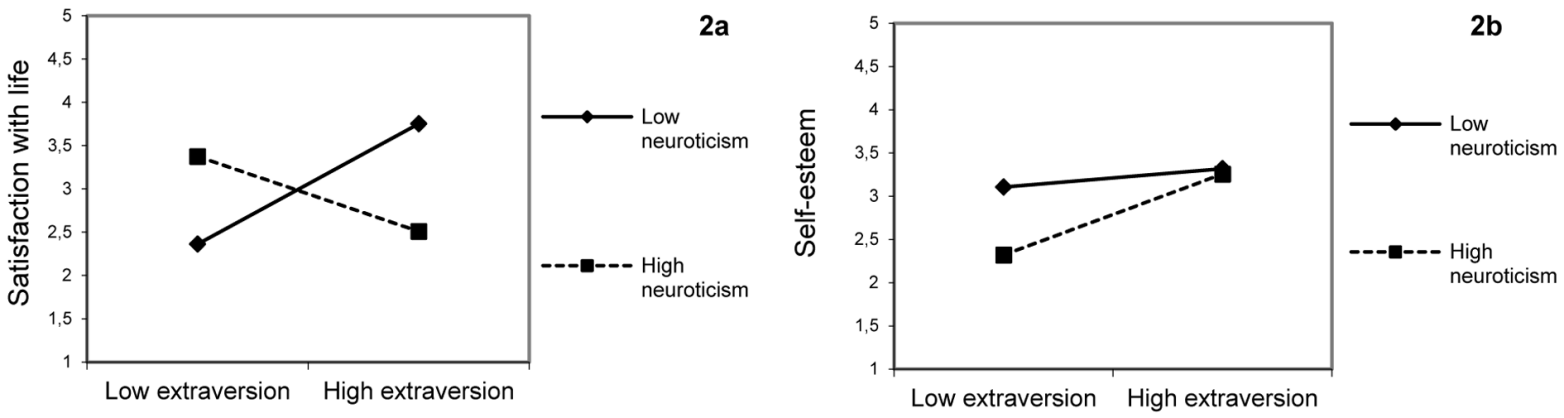
Two-way interaction effects. *Note.* Extraversion range from 1.09 to 4.67 (*M* = 3.29; *SD* = .46); neuroticism range from 1.67 to 4.83 (*M* = 3.20; *SD* = .56).

Concerning Hypothesis a, the interaction between extraversion and neuroticism was statistically significant for Satisfaction with life. The graph reported in Figure 2a shows that the level of neuroticism influences both introverts and extraverts. Consistent with our Hypothesis a1, stable extraverts had a better Satisfaction with life than unstable extraverts; and unstable extraverts showed a pattern of Satisfaction with life similar to stable introverts (both groups are characterized by moderate arousal). Differently from Hypothesis a2, unstable introverts showed high levels of Satisfaction with life.

#### Effects on Self-Esteem

The results showed that extraversion (β = .48; *SE* = .049), neuroticism (β = -.43; *SE* = .051) and their interaction (β = .29; *SE* = .084) influence self-esteem. Figure 2b shows that for low levels of neuroticism, there are no differences between extraverts and introverts. For high levels of neuroticism, extraverts seem to be more comfortable with themselves than introverts.

#### Indirect Effects Mediate by Self-Esteem

As expected (Hypothesis b), the latent interaction variable positively influenced Satisfaction with life and Mastery by an increase in self-esteem (see Table 2). Self-esteem mediated also the relationship between personality traits and SWB. In particular, extraversion (positively) and neuroticism (negatively) affected Satisfaction with life and Mastery through the mediation of self-esteem.

#### Mediated Moderation Effect

To study the mediated moderation effect we examined if self-esteem affects the outcome variables testing a model with Satisfaction with life and Mastery as criterion variables and the interaction term and self-esteem as predictors (Baron & Kenny, 1986). Results showed that self-esteem affects Satisfaction with life (β = .56, *p* < .01) and Mastery (β = .62, *p* < .01). Results showed that self-esteem completely mediates the relationship between the interaction term and Mastery (i.e., the effect of the interaction term on Mastery after controlling for self-esteem is zero), and partially mediates the relationship between the interaction term and Satisfaction with life (the effect of the interaction term on Satisfaction with life after controlling for self-esteem is different from zero: β = -.23, *p* < .01).

#### Moderated Mediation Effect

In line with suggestions provided by Cheung and Lau (2015) and Hayes (2015), we estimated the moderated mediation effect of extraversion on Satisfaction with life and Mastery at various levels of the moderator (neuroticism) to probe the moderated mediation effect. The bootstrap estimated index of moderated mediation was significant for Satisfaction with life (.229, *SE* = .052, 95% bootstrap CI [.156, .359]) and Mastery (.185, *SE* = .034, 95% bootstrap CI [.125, .261]). The results of the mediating effects at various levels of the moderator showed that when neuroticism was at one standard deviation above the mean, the mediating effect of extraversion on SWB dimensions through self-esteem was significant and positive (Satisfaction with life, .348, *SE* = .118, 95% bootstrap CI [.211, .699]), (Mastery, .281, *SE* = .064, 95% bootstrap CI [.191, .472]). When neuroticism was at one standard deviation below the mean, the mediating effect was not significant. These results imply that neuroticism may influence the mediating effect of extraversion on Satisfaction with life and Mastery through self-esteem only when neuroticism is high.

## Discussion and Conclusion

Aim of the present study was to explore the moderating role of neuroticism in the relationship between introversion-extraversion and SWB with and without the possible mediation of self-esteem. Although the relationship between SWB and extraversion, has often been studied in literature, few studies have taken into account the moderating role of neuroticism. Literature suggested that neuroticism might be more important to individual SWB than extraversion and its component of sociability (DeNeve & Cooper, 1998; Hills & Argyle, 2001a, 2001b). Moreover, Young and Bradley (1998) found that unstable introverts may be more likely to suffer from maladjustment than stable introverts.

We have better examined these aspects using a latent framework. Our model is characterized by direct effects of personality traits and self-esteem on SWB dimensions and indirect effects of traits on SWB mediated by self-esteem; moreover, we specifically included in the model a latent interaction term between extraversion and neuroticism.

Our results showed that the nature of differences between introverts and extraverts on SWB could be related to the trait of neuroticism in relation to Satisfaction with life. Stable extravert individuals appear to be more satisfied than the other individuals; this might be the result of some characteristics of the stable extravert, who is a sociable and carefree person. Unstable introvert individuals showed an intermediate level of Satisfaction; whereas the unstable extraverts, often isolated from peers due their aggressive behaviour, showed the lowest level of Satisfaction. Finally, it should also be noted that, unstable extraverts showed pattern of Satisfaction similar to stable introverts. This result is consistent with Eysenck’s theory of the biological bases of personality traits (1967). Indeed, moderate levels of cortical arousal lead both groups to experience similar levels of Satisfaction.

In accordance with previous studies where self-esteem has been reported to be one of the strongest predictors of SWB (e.g., Cheng & Furnham, 2003; Neto, 2001), our results showed that self-esteem had a positive influence on Satisfaction in life and on the overall sense of Mastery, which includes capacities that concur with self-fulfilment, such as life control, making decisions easily, organizing time, and getting started.

Concerning the effects of the interaction term (extraversion x neuroticism) on self-esteem, our results showed that for low levels of neuroticism, there are no differences between extraverts and introverts. In general, stable individuals seem to be comfortable with themselves. This effect changes for high levels of neuroticism. In particular, among introverts, self-esteem is influenced by the level of neuroticism. The stable introverts showed a level of self-esteem higher than the unstable introverts. This is not unexpected considering that the stable introvert is a calm and thoughtful individual, who prefers solitude, intimate relations and feels good about himself when is involved in introspective activities; whereas the unstable introvert has melancholic qualities such as pessimism, anxiety and moody. On the contrary, for individuals with a high level of extraversion, both, stable and unstable extraverts seem to be comfortable with themselves.

In line with suggestions from previous studies on adolescence samples (e.g., Fadda et al., 2015; Furnham & Cheng, 2000), the results of our study support the idea that self-esteem is not only a direct predictor of SWB dimensions related to Satisfaction with life and Mastery, but also a crucial mediator for traits of personality.

In the present study, we found that self-esteem completely mediates the relationship between the interaction term and Mastery, whereas the relationship between the interaction term and Satisfaction with life is only partially mediated by self-esteem. Partial mediation is the case in which the path from the predictors to the outcome is reduced in absolute size but is still different from zero when the mediator is introduced (Baron & Kenny, 1986).

Results of moderated mediation effect analysis showed that, consistent with our second hypothesis, a high level of neuroticism moderates the effect of extraversion on Satisfaction with life and Mastery through the mediation of self-esteem. In general, for unstable introverts and extraverts, being comfortable with themselves seem to positively affect SWB.

The importance of considering the mediation of self-esteem emerged also when we considered separately the traits of extraversion and neuroticism. Indeed, extraversion showed direct influences on dimensions related to energy and vigour required to engage in activities, as well as achievements and fun in social group activities, but not on Satisfaction with life and Mastery. Extraversion appears to promote these SWB dimensions, via an increment of self-esteem. Thus, feeling good about themselves more strongly affects the sense of satisfaction and of mastery, so that adolescents feel in control of the situations; and vice versa if they feel bad about themselves this would greatly detriment their sense of satisfaction and mastery. In a similar way, neuroticism negatively affects self-esteem, which, in turn, decreases the SWB dimensions of Satisfaction with life and Mastery. So feeling bad about themselves would greatly detriment adolescent’s sense of SWB.

One explanation of the positive association between self-esteem and extraversion is that, at least in Western cultures, extraverted tendencies are encouraged and valued more than introverted tendencies (Francis & James, 1996). Other authors have argued that extraversion develops very early and could subsequently contribute to the evolution of other personality-based tendencies (e.g., seeking social support, positive affect), which could serve as potential mediators of the relationship between extraversion and self-esteem (Swickert, Hittner, Kitos, & Cox-Fuenzalida, 2004).

Finally, in the present study, we took into account several SWB dimensions (Satisfaction with life, Mastery, Vigour, Social Cheerfulness). Indeed, SWB is a multifaceted construct, and a multidimensional perspective seems to be appropriate in the study of SWB in adolescence. According to lifespan studies, people’s conceptions of well-being, change with age (Ryff, 1989; Ryff & Singer, 2008). During adolescence, SWB appears to be frequently centered around important personal and social factors, which favour the attainment of the basic goal of autonomy (Ryff & Singer, 1998, 2000).

### Study Limitations

Even though our results await to be further replicated in samples of different ages and culture, they provide interesting insights in the relation between personality traits, self-esteem and SWB. Overall, our hypothesis concerning the moderating role of neuroticism was not confirmed for all dimensions. Although extraversion showed a direct effect on Vigor and Social Cheerfulness, the interaction with neuroticism lead a direct effect only on Satisfaction with life. A possible explanation might be that the dimensions of SWB concerning social relations and energy to engage activities, strongly characterize extraverts and thus are not affected by levels of neuroticism. As for the indirect effects, they concern only the two dimensions of SWB directly affected by self-esteem. Indeed, the interaction term affected, through the mediation of self-esteem, Satisfaction with life (partial mediation) and Mastery (complete mediation). These aspects should be further examined in future studies.

A relevant limitation of the present study relates to direction of causality among the examined constructs. To understand if personality causes SWB or if SWB causes personality it is necessary to use sophisticated methodology like longitudinal designs. In the present investigation, we were confined to a static correlational model. Overall, the question of the direction of causality between personality and SWB is difficult. For example, although many studies showed that self-esteem is a major predictor of SWB, it is also possible that the achievement aspect of happiness could increase individuals’ self-esteem, presumably by making one feel proud of oneself (Argyle & Lu, 1990). From a theoretical point of view, the interactive models of personality, suggest that these relations might be recursive. Nonetheless, concerning traits, some longitudinal and experimental studies (e.g., Magnus, Diener, Fujita, & Pavot, 1993; McNiel & Fleeson, 2006) suggest that extraversion and neuroticism might causally determine SWB. Therefore, this area of research needs to be further examined in future studies.

### Conclusion

In conclusion, the neuroticism trait should be considered, as well as extraversion, a cardinal predictor of SWB. Our results confirmed that the trait of neuroticism moderates the relationships between introversion-extraversion and some SWB dimensions, directly and by mediation of self-esteem. Therefore, future studies interested in SWB personal predictors should take into account not only extraversion but also the level of neuroticism. An important consideration of the present study concerns the relationship between extraversion and Satisfaction with life. Being extravert in association with low neuroticism leads to higher levels of satisfaction. On the contrary, the unstable extraverts are not more satisfied than their introverted peers, both stable and unstable. Finally, we confirm that self-esteem is a factor that can significantly help unstable extraverts and introverts to be happy.
